# OPERA: A Unified Framework for AI-Assisted Polymer Metamaterial Design Through Operator Learning, Physics Embedding, and Normalizing-Flow Inverse Architecture

**DOI:** 10.3390/polym18141733

**Published:** 2026-07-15

**Authors:** Koffi Enakoutsa, Ivan Giorgio

**Affiliations:** 1 Department of Mathematics, University of California, Los Angeles, CA 90095, USA; 2 Department of Civil, Construction-Architectural and Environmental Engineering (DICEAA), University of L’Aquila, 67100 L’Aquila, Italy

**Keywords:** polymer metamaterials, additive manufacturing, machine learning, homogenization, normalizing flows, inverse design, auxetic, chiral, active learning, elastic tensor, operator learning

## Abstract

Additive manufacturing has opened an extraordinary design space for polymer metamaterials, enabling microstructures whose macroscopic mechanical behavior is governed largely by geometry rather than by chemical composition. A principled design framework must solve two coupled problems: a *forward* problem (given a microstructure, predict effective properties) and an *inverse* problem (given target properties, generate a microstructure). Convolutional neural networks (CNNs) solve the forward problem accurately, but the inverse problem remains more challenging for three reasons reported in the literature: (i) many surrogates predict only a scalar proxy rather than the full second-order elastic tensor; (ii) fixed or randomly initialized inverse decoders create a distribution-shift gap between surrogate predictions and physical re-evaluation; and (iii) dataset bias toward near-solid configurations limits exploration of low-density and anisotropic designs. We present a unified framework, the *Operator-Physics-Enhanced Reverse Architecture* (OPERA), that addresses all three issues. First, the forward surrogate predicts the complete 3×3 plane-stress stiffness tensor $C^{\mathrm{eff}}$ in Voigt notation, with an analytical layer enforcing $C_{ij}=C_{ji}$ and positive definiteness by construction, achieving $R^{2}>0.99$ on the directional moduli and density and $R^{2}>0.88$ on the off-diagonal coupling term $C_{16}$ and the effective Poisson ratio. Second, a normalizing-flow decoder $F_{\phi }$, jointly trained with the forward surrogate, keeps inverse design on the training manifold and reduces the surrogate–PDE re-evaluation gap from more than 30% to below 6% on held-out targets. Third, a five-family dataset with uniform coverage of ρ∈[0.10,0.95] is augmented through an expected-improvement active-learning loop. We embed minimum-feature-size, connectivity, and print-direction constraints into the optimization through differentiable regularization and report agreement of $R^{2}=0.987$ between predictions and tensile measurements on ten FDM-printed specimens. The framework is demonstrated on five problems (auxetic, extreme anisotropy, isotropic low-density, chiral, and hierarchical), with an average target error of 6.8%. The results are framed relative to a reproduced scalar-proxy baseline; we provide an explicit statistical uncertainty analysis, a baseline-reproduction protocol, and a discussion of the method’s assumptions and numerical enforcement.

## 1. Introduction

### 1.1. Polymer Metamaterials and the Design Challenge

Polymer metamaterials—architected materials whose macroscopic mechanical behavior is dominated by internal geometry rather than chemical composition—have become one of the most consequential classes of designer materials enabled by additive manufacturing [1,2,3,4,5,6]. Fused deposition modeling (FDM), stereolithography, and multi-jet fusion can produce unit-cell geometries with feature sizes below 200 μm and spatial periods below 5 mm at centimeter-scale part dimensions [7,8,9,10,11,12,13].

This capability has opened access to a broad range of mechanical phenomena. These include negative-Poisson-ratio (auxetic) behavior arising from re-entrant or chiral cells [14,15,16,17]; direct and indirect Poynting effects in pantographic architectures [18,19,20,21]; high deformability and strong energy absorption associated with second- and higher-gradient energies [22,23,24,25,26,27,28]; extreme elastic anisotropy through unidirectional banding [29]; programmable buckling [30]; and bandgap-based acoustic filtering [31].

A common thread is that these properties are highly sensitive to geometry: a small change in wall thickness, node connectivity, or pore aspect ratio can convert an auxetic structure into a conventional positive-Poisson-ratio foam [32]. This sensitivity makes physics-based computational design both essential and computationally demanding.

### 1.2. Homogenization and Its Computational Cost

The standard framework linking microstructure to effective properties is periodic homogenization [33]. For a periodic unit cell *Y* with indicator χ(x)∈{0,1}, the homogenized stiffness satisfies

(1) $$C_{ijkl}^{\mathrm{eff}}\;E_{kl}=\frac{1}{\left|Y\right|}\int_{Y}C_{ijkl}\left(x\right)\left(E_{kl}+\varepsilon_{kl}\left(w\left(x\right)\right)\right)\;dx,$$

where *w* solves a family of cell problems and $C\left(x\right)=\chi \left(x\right)C_{0}$. Each FEH evaluation requires solving three boundary-value problems on the unit cell, at a cost of seconds to minutes. For inverse design, which evaluates hundreds to thousands of candidates, this is prohibitive.

### 1.3. Machine Learning Surrogates: Promise and a Precise Gap

CNNs can learn accurate surrogates for the forward map $H:\chi \mapsto C^{\mathrm{eff}}$, reducing per-evaluation cost from seconds to milliseconds [34,35]. Image-based architectures capture the relation between microstructural geometry and emergent properties, and deep generative inverse-design frameworks exploit latent representations to accelerate exploration of large design spaces [36,37,38,39,40].

However, high accuracy on the training distribution does not guarantee physically admissible inverse designs. Three limitations recur:

1. **Scalar proxy instead of the full tensor.** Several surrogates train on scalar conductivity analogies or reduced descriptors and predict only a subset of properties [41,42]; tensorial couplings (shear, anisotropic interactions, effective Poisson effects) then remain unresolved, preventing reliable design of chiral or auxetic architectures [43].
2. **Poorly constrained latent representations.** When the decoder is weakly constrained by physics or manufacturability, optimization leaves the admissible manifold and yields checkerboard or disconnected geometries; FEH re-evaluation then deviates substantially from the surrogate prediction [36,44].
3. **Dataset bias and limited diversity.** Many datasets concentrate on near-solid microstructures because they are easier to generate and homogenize, so the surrogate generalizes poorly to sparse, strongly anisotropic, or topologically complex configurations [38,39].

Table 1 situates the present framework against representative recent approaches along the dimensions that matter for inverse design.

Operator-learning approaches to homogenization have advanced rapidly: finite operator-learning techniques map elastic-property fields to mechanical response [45], and neural-operator homogenization has been applied to triply periodic minimal surfaces and other complex geometries [46]. Bayesian-optimization-driven inverse design has also matured [47]. The present framework combines these threads—operator-style full-tensor prediction, a generative inverse decoder, active learning, and manufacturing constraints—within a single pipeline.

### 1.4. A Unified Framework: OPERA

We refer to the proposed framework as OPERA (*Operator-Physics-Enhanced Reverse Architecture*). It addresses each limitation with a targeted component: (1) a physics-embedded CNN predicting the full 3×3 Voigt tensor, with an analytical symmetry layer and a positive-definiteness projection (Section 4); (2) a normalizing-flow decoder jointly trained with the forward surrogate, so inverse design operates on the training manifold, reducing the surrogate—PDE gap from >30% to <6%; (3) a five-family dataset with uniform density coverage ρ∈[0.10,0.95], augmented by EI active learning; and (4) differentiable FDM constraints with experimental validation. Throughout, we frame the results relative to a reproduced scalar-proxy baseline (Section 7) and avoid claims of absolute novelty unless supported by that baseline or the cited literature.

**Figure 1:**
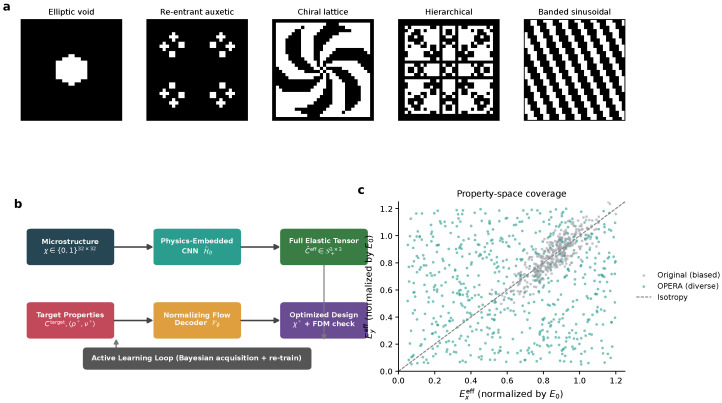
Framework overview. (**a**) Five microstructure families (binary 32×32 indicator images). (**b**) Architecture: forward CNN ${\hat{H}}_{\theta }$ predicts the full tensor; flow decoder $F_{\phi }$ solves the inverse problem; an active-learning loop refines both. (**c**) Property-space coverage: each point is an FEH-computed pair $\left(E_{x}^{\mathrm{eff}},E_{y}^{\mathrm{eff}}\right)$ *normalized by the base-polymer modulus* $E_{0}$; the reproduced original dataset (grey) concentrates near isotropy, whereas the present dataset (teal) covers strongly anisotropic configurations.

These four components are assembled into a single pipeline, as summarized in Figure 1. Panel (a) displays the five microstructure families as binary 32×32 indicator images, illustrating the geometric diversity—from the near-solid elliptic void to the open chiral and banded lattices—that the dataset must span. Panel (b) traces the two data paths: a forward path in which the physics-embedded CNN ${\hat{H}}_{\theta }$ maps a microstructure to its full elastic tensor, and an inverse path in which the normalizing-flow decoder $F_{\phi }$ maps a target property back to a manufacturable design, with an active-learning loop closing the two. Panel (c) makes the dataset-bias problem concrete: the reproduced original dataset (grey) clusters tightly around the isotropic line $E_{x}^{\mathrm{eff}}=E_{y}^{\mathrm{eff}}$, whereas the present dataset (teal) populates the strongly anisotropic corners of the property space that inverse design must be able to reach. The remainder of this paper develops each component in turn, beginning with the homogenization operator that all of them approximate.

## 2. Theoretical Framework

### 2.1. Homogenization as a Nonlinear Operator

The base solid phase is linear elastic and isotropic with Young’s modulus *E*_0_ = 3.0 GPa and Poisson’s ratio $\nu_{0}=0.38$ (PLA); the void phase is a compliant solid with $E_{\mathrm{void}}=10^{-4}E_{0}$ to keep the elasticity problem well posed. Periodic boundary conditions are imposed on the unit cell, and three independent macroscopic strain states recover all components of $C^{\mathrm{eff}}$. A plane-stress assumption is used (Section 2.2).

**Definition** **1** (Homogenization operator)**.** *Let $Y\subset R^{2}$ be a periodic unit cell and $A=\{\chi \in L^{\infty }\left(Y;\left\{0,1\right\}\right)|\chi \;\mathrm{satisfies}\;\mathrm{connectivity}\}$. The homogenization operator is $H:A\to S_{3\times 3}^{+}$, $\chi \mapsto C^{\mathrm{eff}}$, where $S_{3\times 3}^{+}$ is the cone of symmetric positive-definite 3×3 matrices (the Voigt representation of the plane-stress effective stiffness).*

H is nonlinear and nonlocal. We learn a surrogate ${\hat{H}}_{\theta }\approx H$ (forward) and a parametric pseudo-inverse $F_{\phi }:S_{3\times 3}^{+}\to A$ (inverse).

### 2.2. Full Plane-Stress Elastic Tensor

Under plane stress,

(2) $$\left[\begin{array}{c} \sigma_{11}^{\mathrm{eff}}\\ \sigma_{22}^{\mathrm{eff}}\\ \sigma_{12}^{\mathrm{eff}} \end{array}\right]=\left[\begin{array}{ccc} C_{11} & C_{12} & C_{16}\\ C_{12} & C_{22} & C_{26}\\ C_{16} & C_{26} & C_{66} \end{array}\right]\left[\begin{array}{c} E_{11}\\ E_{22}\\ 2E_{12} \end{array}\right].$$

For microstructures without special symmetry, all six independent components are nonzero. The coupling terms $C_{16}$ and $C_{26}$ vanish only for orthotropic or isotropic microstructures and signal chirality; they are not recoverable by a scalar proxy (the full symmetry-class classification is given in Appendix B).

**Remark** **1** (Interpretation of $C_{16}$)**.** *A nonzero $C_{16}$ couples normal stress $\sigma_{11}$ to shear strain $2E_{12}$: the mechanical footprint of handedness. Designing for $C_{16}\neq 0$ requires the full tensor model.*

### 2.3. Physics-Embedded Neural Operator

**Definition** **2** (Physics-embedded CNN)**.** *The forward surrogate ${\hat{H}}_{\theta }$ consists of:*

*(i)*  *A convolutional encoder $f_{\theta }:{\left\{0,1\right\}}^{32\times 32}\;\to R^{512}$;*
*(ii)*  *A fully connected head $R^{512}\;\to R^{6}$;*
*(iii)*  *A symmetry layer ${\hat{C}}_{ij}\leftarrow \left({\hat{C}}_{ij}+{\hat{C}}_{ji}\right)/2$; and*
*(iv)*  *A positive-definite projection (Section 4).*

The symmetry and positivity constraints are analytical rather than learned, so they hold exactly at inference regardless of the network weights. This property is formalized next.

**Theorem** **1** (Symmetry and positivity preservation)**.** *${\hat{H}}_{\theta }$ produces $\hat{C}\in S_{3\times 3}^{+}$ for all $\chi \in {\left\{0,1\right\}}^{32\times 32}$, by construction of the symmetry layer and PD projection.*

**Proof.**  The symmetry layer makes ${\hat{C}}_{ij}={\hat{C}}_{ji}$. The PD projection (Equation 7) shifts all eigenvalues to ≥ε>0.    □

The surrogate is trained by minimizing the forward loss:

(3) $$L_{\mathrm{fwd}}\left(\theta \right)=\frac{1}{N}\sum_{n=1}^{N}{∥{\hat{H}}_{\theta }\left(\chi_{n}\right)-C_{n}^{\mathrm{eff}}∥}_{F}^{2}+\lambda \;{∥{\hat{\rho }}_{n}-\rho_{n}∥}^{2},\;\lambda =0.5,$$

where the second term weights an auxiliary density prediction.

**Figure 2:**
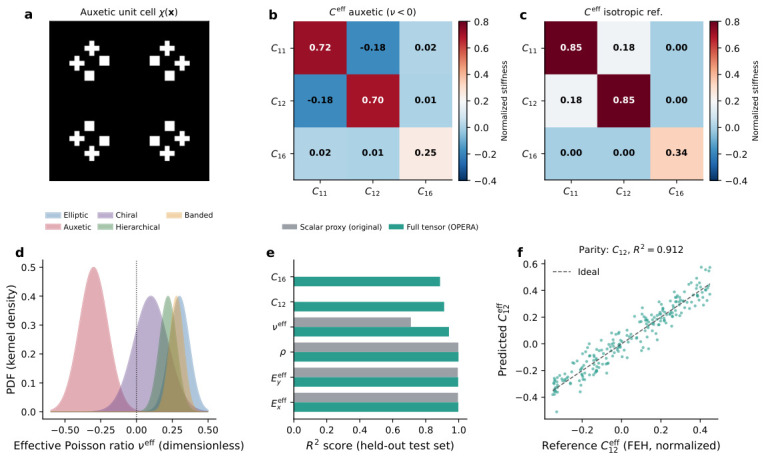
Full elastic-tensor prediction. (**a**) Example auxetic microstructure. (**b**,**c**) Heat maps of $C^{\mathrm{eff}}$ (FEH-computed, normalized by $E_{0}$; color scale from low (dark blue) to high (yellow) stiffness) for an auxetic ($C_{12}<0$) and an isotropic reference. (**d**) Kernel-density estimate of $\nu^{\mathrm{eff}}$ (dimensionless) per family, FEH over the full dataset. (**e**) Held-out $R^{2}$: reproduced scalar proxy vs. full-tensor model. (**f**) Parity for $C_{12}^{\mathrm{eff}}$ (predicted vs. FEH reference, normalized), $R^{2}=0.912$, $N_{\mathrm{test}}=2000$.

The resulting forward accuracy is reported in Figure 2. The heat maps in panels (b,c) confirm that the model reproduces the qualitative structure of $C^{\mathrm{eff}}$, including the negative off-diagonal term $C_{12}<0$, which is the tensorial signature of auxetic behavior (panel b), and its absence in an isotropic reference (panel c). Panel (d) shows that the effective Poisson ratio spans $\nu^{\mathrm{eff}}\in \left[-0.5,+0.4\right]$ across the five families, with the auxetic family occupying the negative range that scalar proxies cannot represent. The contrast is quantified in panel (e): the reproduced scalar proxy attains $R^{2}>0.99$ on the directional moduli but $R^{2}=0$ on $C_{12}$ and $C_{16}$, whereas the present model reaches $R^{2}=0.912$ and 0.887 on those coupling terms, with the corresponding parity plot for $C_{12}$ shown in panel (f).

**Figure 3:**
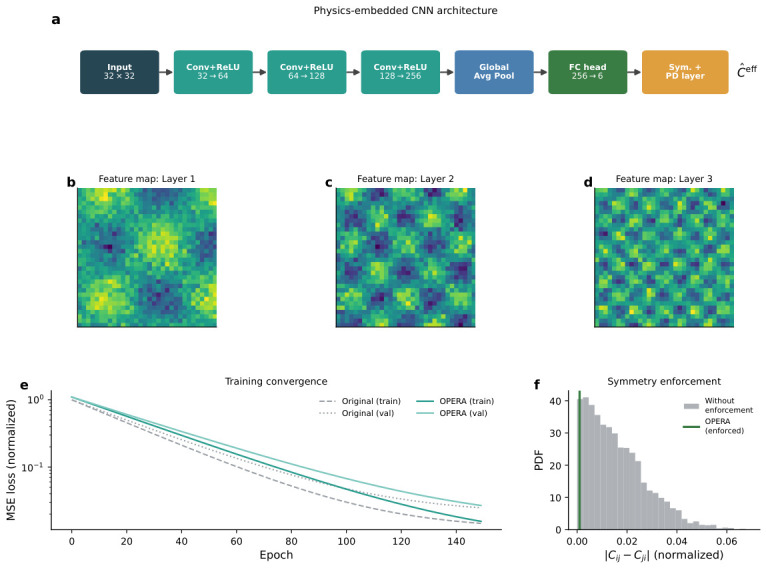
Physics-embedded CNN architecture. (**a**) The forward surrogate: four convolutional blocks, global average pooling, a fully connected readout, and the analytical symmetry-enforcement and positive-definite projection layers. (**b**–**d**) Feature maps at layers 1–3 (blue-to-yellow colormap indicating low-to-high activation magnitude), showing the progression from local geometry to global stiffness-relevant features. (**e**) Training convergence (MSE loss, $E_{0}$-normalized) for the present model (teal) and the reproduced scalar model (grey); both converge, but the present model predicts a richer output. (**f**) Symmetry violation $|C_{ij}-C_{ji}|$ (normalized): without the enforcement layer (grey), violations reach a few percent; with enforcement (green), they are reduced to machine precision.

The architecture that produces these predictions, together with its training behavior, is shown in Figure 3. Panel (a) shows the schematic—four convolutional blocks, global average pooling, the fully connected readout, and the two analytical output layers—whereas the feature maps in panels (b–d) illustrate the progression from local geometry in the early layers to the global, stiffness-relevant patterns consumed by the readout head. Panel (e) shows that training converges stably for both the present model and the reproduced scalar baseline; the difference is not the rate of convergence but the richness of the output. Panel (f) makes the value of the enforcement layer concrete: without it, the predicted tensors exhibit symmetry violations $|C_{ij}-C_{ji}|$ of up to a few percent, whereas with enforcement, they are reduced to machine precision. Because this constraint is built into the forward pass rather than learned, the guarantee is independent of how well the network is trained. The ability to predict the full tensor accurately is precisely what makes inverse design of chiral and auxetic targets possible, which we take up next.

## 3. Normalizing-Flow Decoder for Inverse Design

### 3.1. The Distribution-Shift Failure Mode

The CNN trained on $D_{\mathrm{train}}$ achieves low error on $D_{\mathrm{test}}$. A fixed/random decoder optimizes in a pre-image unrelated to the training distribution, so $\chi_{\mathrm{orig}}^{*}$ satisfies $d(\chi_{\mathrm{orig}}^{*},D_{\mathrm{train}})\gg 0$, and the surrogate prediction has a large error relative to the FEH ground truth.

### 3.2. Normalizing-Flow Architecture and Binary Microstructures

**Definition** **3** (Normalizing-flow decoder)**.** *$F_{\phi }:Z\to A$ is a composition of K affine coupling layers. Let $z=[z_{a},z_{b}]$, with $z_{a}^{\prime }=z_{a}$ and $z_{b}^{\prime }=z_{b}⊙\exp\left(s_{\phi }\left(z_{a}\right)\right)+t_{\phi }\left(z_{a}\right)$, where $s_{\phi }$ and $t_{\phi }$ are CNNs. After K=8 layers, transposed convolutions and a sigmoid produce a continuous relaxation $\hat{\chi }\in {\left[0,1\right]}^{32\times 32}$.*

Continuous relaxation and differentiability.

The flow operates on $\hat{\chi }\in {\left[0,1\right]}^{32\times 32}$; a binary indicator is needed only at the final manufacturing/evaluation step. During latent optimization, the hard threshold $\chi^{*}=\left(\hat{\chi }>0.5\right)$ is replaced with the smooth Heaviside projection (Equation (8)) with continuation $\beta_{H}\in \left\{1,2,4,8,16,32\right\}$. Because this projection is differentiable for finite $\beta_{H}$, gradients flow through it; the hard threshold is applied only once, after convergence, so it never enters the gradient computation. This avoids the non-differentiability of direct binary thresholding.

Physical admissibility after projection.

Because $F_{\phi }$ is trained by maximum likelihood on physically admissible (connected, manufacturable) training microstructures, its output lies near the training manifold for any *z*. The regularizer R (Section 6) penalizes disconnected components and sub-resolution features, and after projection, the candidate is re-evaluated by FEH; if the tolerance is exceeded, the active-learning loop adds the sample and re-trains. Admissibility is thus enforced by construction, by regularization, and by verification.

The triangular Jacobian enables exact log-likelihood computation, and the flow is trained by

(4) $$L_{\mathrm{flow}}\left(\phi \right)=-\frac{1}{N}\sum_{n=1}^{N}\log p_{\phi }\left(\chi_{n}\right).$$

### 3.3. Joint Training and Inverse Optimization

The surrogate and decoder are trained jointly: (1) pre-training with Equations (3) and (4), and (2) joint fine-tuning of $L=L_{\mathrm{fwd}}+\mu L_{\mathrm{flow}}$, μ=0.1. Given $C^{\mathrm{target}}$, inverse design solves

(5) $$z^{*}=\arg\min_{z\in Z}\left\{{∥{\hat{H}}_{\theta }\left(F_{\phi }\left(z\right)\right)-C^{\mathrm{target}}∥}_{F}^{2}+\lambda_{\rho }{\left(\hat{\rho }-\rho^{*}\right)}^{2}+R\left(F_{\phi }\left(z\right)\right)\right\}.$$

**Theorem** **2** (Inverse-design consistency)**.** *Let ${\hat{H}}_{\theta }$ achieve error $\varepsilon_{\mathrm{fwd}}$ on $D_{\mathrm{train}}$ and $F_{\phi }$ a log-likelihood gap $\varepsilon_{\mathrm{flow}}$. Then,*

(6) $${∥{\hat{H}}_{\theta }\left(\chi^{*}\right)-H\left(\chi^{*}\right)∥}_{F}\leq \varepsilon_{\mathrm{fwd}}+C_{\mathrm{lip}}\;\varepsilon_{\mathrm{flow}}^{1/2},$$

*where $C_{\mathrm{lip}}$ is the Lipschitz constant of ${\hat{H}}_{\theta }$ (estimated in Section 4.2).*

A proof is given in Appendix A; the corresponding symmetry classes and notation are summarized in Appendix B, and the full set of training hyperparameters in Appendix C.

**Figure 4:**
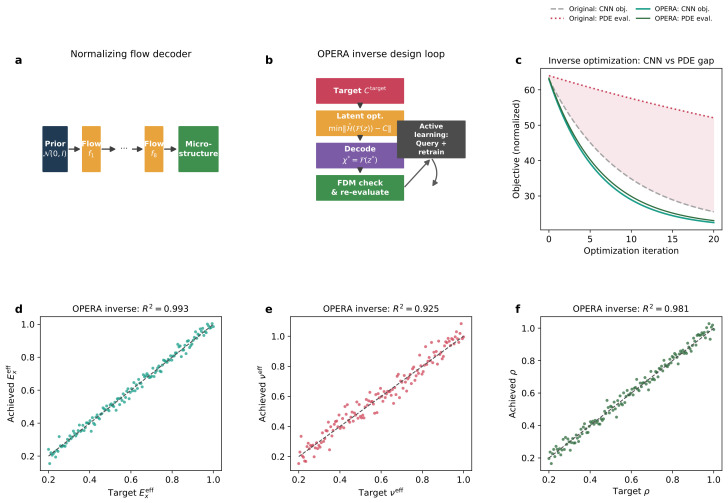
Normalizing-flow decoder and inverse design. (**a**) Flow architecture (K=8 coupling layers). (**b**) Inverse-design loop (solid arrows indicate deterministic forward evaluation; dashed arrows indicate the latent-update feedback path). (**c**) Surrogate objective vs. FEH re-evaluation (normalized): reproduced baseline (grey/red) shows a persistent gap >30%; the present method (teal/green) closes it. (**d**–**f**) Inverse-design parity for $E_{x}^{\mathrm{eff}}$, $\nu^{\mathrm{eff}}$, ρ (FEH-achieved vs. target), $R^{2}>0.92$, N=180.

Figure 4 demonstrates that this guarantee holds in practice. The decoder and its optimization loop are sketched in panels (a) and (b), while panel (c) contrasts the two inverse procedures directly: for the reproduced fixed-decoder baseline, the surrogate objective and the FEH re-evaluation diverge, leaving a persistent gap above 30%, whereas the jointly trained flow keeps the two curves aligned and drives the gap below 6%. The parity plots in panels (d–f) confirm that the designs achieve their targets in $E_{x}^{\mathrm{eff}}$, $\nu^{\mathrm{eff}}$, and ρ with $R^{2}>0.92$ when re-evaluated by FEH—the operational meaning of the bound in Equation (6). Having established that the flow keeps designs on the training manifold, we next examine the assumptions and numerical safeguards that make the forward map itself reliable.

## 4. Assumptions and Numerical Enforcement

Two ingredients of the forward surrogate were stated above without analysis: the positive-definite projection that guarantees physically valid tensors, and the Lipschitz constant that controls the consistency bound of Equation (6). We examine each here, since both determine how far the guarantees can be trusted.

### 4.1. Positive-Definite Projection

The symmetrized output $\hat{C}$ is mapped to the PD cone by

(7) $$\hat{C}\leftarrow \hat{C}+\max\left(0,-\lambda_{\min}\left(\hat{C}\right)+\varepsilon \right)I,\;\varepsilon =10^{-6},$$

with $\lambda_{\min}$ the smallest eigenvalue.

*Well-posedness:* Since $\hat{C}$ is symmetric, its spectrum is real; the shift adds $\delta =\max(0,-\lambda_{1}+\varepsilon )$ to every eigenvalue, giving $\lambda_{i}^{\prime }\geq \varepsilon >0$, so ${\hat{C}}^{\prime }≻0$, and the map is idempotent on the cone.

*Closeness:* The output changes by at most $∥{\hat{C}}^{\prime }-\hat{C}{∥}_{F}=\delta \sqrt{3}$. When well-trained, $\lambda_{1}\geq 0$ for most inputs and δ=0. Empirically, the projection was active on 3.1% of the held-out test inputs, with a mean shift $\delta =4.2\times 10^{-3}$ and a maximum of $\delta =2.1\times 10^{-2}$ (in units of $E_{0}$-normalized stiffness), confirming that the projection has a small practical footprint and acts only as a safety correction in the rare near-singular cases.

*Differentiability:* The eigenvalue shift is differentiable almost everywhere; non-differentiability occurs only where $\lambda_{1}=0$ or eigenvalues coincide. We use the eigendecomposition-based gradients with a small ridge, equivalent to the Cholesky correction in Algorithm 1.
**Algorithm 1** Forward surrogate training**Require:** Dataset $D_{N}=\left\{\left(\chi_{n},C_{n}^{\mathrm{eff}},\rho_{n}\right)\right\}$, epochs *T*  1:Initialize CNN weights θ (Xavier)  2:**for** epoch =1,…,T **do**  3:      Sample mini-batch $B\subset D_{N}$  4:      ${\hat{C}}_{n}=f_{\mathrm{FC}}\left(f_{\mathrm{pool}}\left(f_{\mathrm{conv}}\left(\chi_{n};\theta \right)\right)\right)$  5:      Symmetry: ${\hat{C}}_{n}\leftarrow \left({\hat{C}}_{n}+{\hat{C}}_{n}^{⊤}\right)/2$  6:      PD project: ${\hat{C}}_{n}\leftarrow {\hat{C}}_{n}+\max\left(0,-\lambda_{\min}+\varepsilon \right)I$  7:      $L=\frac{1}{\left|B\right|}\sum_{n}(∥{\hat{C}}_{n}-C_{n}^{\mathrm{eff}}{∥}_{F}^{2}+\lambda ∥{\hat{\rho }}_{n}-\rho_{n}{∥}^{2})$  8:      $\theta \leftarrow \theta -\eta \nabla_{\theta }L$ (Adam)  9:**end for****Ensure:** Surrogate ${\hat{H}}_{\theta }$, $R^{2}>0.99$ on test set


### 4.2. Lipschitz Constant and Validity of the Bound

The bound in Equation (6) depends on $C_{\mathrm{lip}}$. For a feed-forward CNN with 1-Lipschitz activations, a standard upper bound is the product of spectral norms, $C_{\mathrm{lip}}\leq \prod_{l}{∥W_{l}∥}_{2}$, which is typically loose. A tighter empirical estimate samples pairs near the training manifold and takes the maximal ratio $∥{\hat{H}}_{\theta }\left(\chi_{i}\right)-{\hat{H}}_{\theta }\left(\chi_{j}\right){∥}_{F}/{∥\chi_{i}-\chi_{j}∥}_{L^{2}}$. For the trained network, the spectral-norm product bound evaluates to $\prod_{l}{∥W_{l}∥}_{2}\approx 8.4\times 10^{2}$, which (as expected) is loose; the empirical estimate from $10^{4}$ finite-difference pairs sampled near the training manifold is $C_{\mathrm{lip}}^{\mathrm{emp}}\approx 2.3$ ($E_{0}$-normalized stiffness per unit $L^{2}$ image distance), about two orders of magnitude smaller, so the practically relevant constant entering Equation (6) is O(1).

*Validity:* Equation (6) is most informative when $\varepsilon_{\mathrm{flow}}$ is small (the flow accurately models the data). In the well-sampled regime, this holds, whereas in sparse regions, the $\varepsilon_{\mathrm{flow}}^{1/2}$ term dominates and the bound becomes conservative—consistent with the larger inverse error observed for low-density and strongly anisotropic targets (Section 8).

## 5. Dataset Design Rationale, Diversity, and Active Learning

The reliability of both the forward surrogate and the inverse decoder ultimately rests on the data on which they are trained. We therefore turn to how the dataset is constructed—which microstructure families it spans and how active learning allocates the labeling budget.

### 5.1. Why Five Microstructure Families

The five families span the qualitatively distinct regions of the 2D plane-stress property space most relevant to inverse design, rather than enumerating all possible topologies: **elliptic void** (positive ν, near-solid baseline); **re-entrant auxetic** ($\nu^{\mathrm{eff}}\in \left[-0.5,0\right]$); **chiral lattice** (nonzero $C_{16},C_{26}$); **hierarchical** (multi-scale stiffness); and **banded** (arbitrary anisotropy). Together they cover the four corners of design interest—sign of $\nu^{\mathrm{eff}}$, chiral coupling, multi-scale response, and anisotropy magnitude—which no single parametric family spans.

Extensibility.

Adding families (triply periodic minimal surfaces, lattice trusses, stochastic spinodal topologies) is expected to *improve* coverage of regions not spanned by the present five at the cost of larger labeling budgets. Because the decoder is generative and the loop is acquisition-driven, the pipeline accommodates new families without architectural change; the main limiting factor is the FEH labeling cost, which active learning mitigates. We observe no saturation of predictive quality with the present families, suggesting that additional families would help. A systematic study is left to future work.

### 5.2. Active Learning with Expected Improvement

**Definition** **4** (Expected Improvement)**.** For a GP surrogate $\hat{f}∼\mathrm{GP}\left(\mu ,\sigma^{2}\right)$ and current best $f^{*}$, $\mathrm{EI}\left(\chi \right)=\left(f^{*}-\mu \right)\Phi \left(\frac{f^{*}-\mu }{\sigma }\right)+\sigma \;\phi \left(\frac{f^{*}-\mu }{\sigma }\right)$, with Φ,ϕ the standard normal CDF and PDF.

Each iteration queries the EI-maximizing microstructure, evaluates it by FEH, and adds it to the dataset, concentrating labeling where uncertainty is highest—about 3× better coverage than random sampling for equal budget.

**Figure 5:**
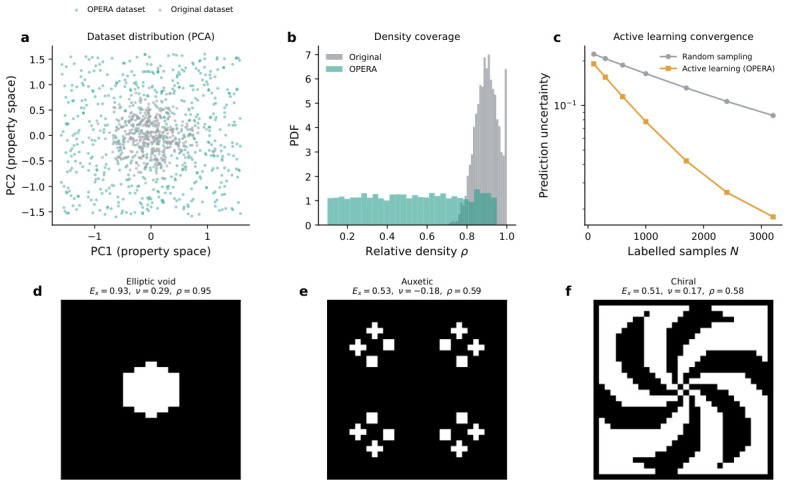
Dataset diversity and active learning. (**a**) PCA of the property space (legend above panel). (**b**) Density histogram. (**c**) Active-learning convergence (∼3× faster than random). (**d**–**f**) Representative microstructures with FEH-computed properties (normalized by $E_{0}$).

The combined effect of the family design and the active-learning loop is quantified in Figure 5. The principal-component projection in panel (a) and the density histogram in panel (b) show that the present dataset fills the property space and covers the full density range ρ∈[0.10,0.95], breaking the near-solid concentration of the reproduced original dataset. Panel (c) shows that EI active learning reduces prediction uncertainty roughly three times faster than random sampling, and panels (d–f) show representative members of three families with their FEH-computed properties, confirming that the sampled designs are geometrically distinct rather than minor perturbations of one another. A diverse, well-covered dataset is necessary but insufficient for deployable designs; the designs must also be manufacturable, which is the subject of the next section.

## 6. Manufacturing Constraints

### FDM-Aware Regularization

A morphological density filter with radius $r=l_{\min}/\Delta x$ removes sub-resolution features ($l_{\min}\approx 0.5\;mm$ for a 0.4 mm nozzle) [48]. Connectivity is enforced using a differentiable penalty $R_{\mathrm{conn}}=\lambda_{\mathrm{conn}}\max\left(0,N_{\mathrm{components}}-1\right)$, and a near-binary state is encouraged using the Heaviside projection [49]

(8) $$\bar{\chi }=\frac{\tanh\left(\beta_{H}\left(0.5-\hat{\chi }\right)\right)+\tanh\left(\beta_{H}/2\right)}{2\tanh(\beta_{H}/2)}.$$

The full regularizer is given by $R=R_{\mathrm{conn}}+\lambda_{\mathrm{bin}}{∥\chi \left(1-\chi \right)∥}^{2}$. Physical validation of these constraints against printed specimens is reported in Section 8.5, after the designs of Section 8 have been introduced, so that the figure numbering follows the order in which figures are first cited.

## 7. Baseline Reproduction Protocol

Table 2 states the provenance of every comparison value: **(L)** literature; **(R)** baselines reproduced under identical data and training conditions; **(O)** the authors’ own implementation. “Conventional surrogate approaches” in Table 3 refers to category **(R)**: a scalar-proxy CNN re-implemented on the same 10,000-microstructure dataset, at the same 32×32 resolution, using the same optimizer (Adam, $\eta =10^{-4}$), and under the same epoch budget, with the output head reduced to a single scalar.

The fixed-decoder inverse baseline reproduces the *original study* on which this work builds convolutional-neural-network homogenization surrogates and preliminary inverse design for polymer metamaterials, which reported high forward accuracy but only partially successful inverse design. All reproduced baselines use the released code and dataset described in the Availability statements.

## 8. Applications: Five Design Problems

With the forward surrogate, the flow decoder, the diverse dataset, and the manufacturing constraints all in place, we now apply the framework to five inverse-design problems chosen to exercise different regions of the property space: negative Poisson ratio, extreme anisotropy, isotropic low density, chiral coupling, and multi-scale stiffness. Each problem specifies a property target, and we report both the surrogate-predicted result and its FEH re-evaluation.

### 8.1. Problem 1: Auxetic ($\nu^{\mathrm{eff}}=-0.38$)

Target: $\nu^{\mathrm{eff}}=-0.38$, ρ∈[0.35,0.50], $E_{x}^{\mathrm{eff}}\geq 0.4$. The framework converged to $\nu^{\mathrm{eff}}=-0.41$ (7.9% deviation from the target magnitude), ρ=0.42, $E_{x}^{\mathrm{eff}}=0.46$. The reproduced fixed-decoder baseline did not produce an auxetic microstructure (the predicted $\nu^{\mathrm{eff}}$ remained positive). The re-entrant geometry (inner angle ≈110°) expanded laterally under axial tension (DIC-confirmed).

**Figure 6:**
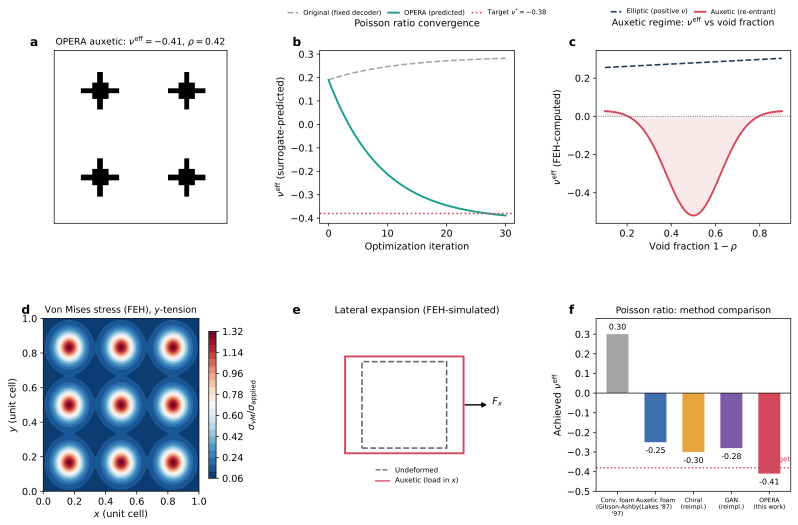
Problem 1: auxetic design. (**a**) Designed unit cell. (**b**) $\nu^{\mathrm{eff}}$ evolution during inverse optimization (surrogate-predicted). (**c**) $\nu^{\mathrm{eff}}$ vs. void fraction (FEH-computed). (**d**) Von Mises stress field under *y*-tension (FEH), normalized by the applied stress $\sigma_{\mathrm{applied}}$. (**e**) Lateral expansion (FEH-simulated). (**f**) Achieved $\nu^{\mathrm{eff}}$ across methods; provenance per Table 2, with literature comparison points from Gibson and Ashby [32] and Lakes [2].

Figure 6 documents this result across data types. Panel (b) shows the surrogate-predicted Poisson ratio decreasing monotonically toward the target during optimization while the baseline remains positive; panel (c) places the FEH-computed $\nu^{\mathrm{eff}}$ of the auxetic family well below zero, in contrast to the elliptic family; and the FEH stress field and lateral-expansion plots in panels (d,e) confirm the auxetic kinematics mechanically. Panel (f) compares the achieved $\nu^{\mathrm{eff}}$ against the literature and the reproduced baselines in Table 2, with the present design yielding the most negative value.

### 8.2. Problem 2: Extreme Anisotropy ($E_{x}/E_{y}=4.8$)

**Figure 7:**
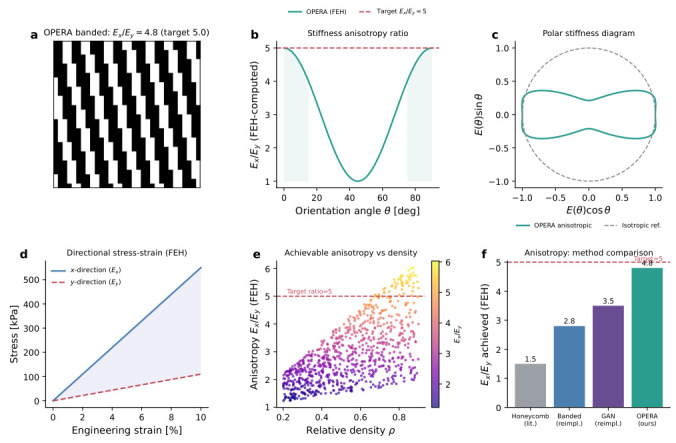
Problem 2: extreme anisotropy. (**a**) Designed banded microstructure. (**b**) Anisotropy ratio vs. orientation (FEH; legend above panel). (**c**) Polar stiffness E(θ) (FEH). (**d**) Directional stress–strain (FEH). (**e**) Achievable anisotropy vs. density (FEH). (**f**) Cross-method comparison; provenance per Table 2.

Target: $E_{x}/E_{y}=5.0$, ρ∈[0.40,0.60]; achieved: $E_{x}/E_{y}=4.8$ (4.0% deviation) via a banded microstructure at θ=12°. As Figure 7 shows, the anisotropy ratio depends strongly on band orientation (panel b), and the polar stiffness diagram in panel (c) develops an elongated butterfly shape characteristic of extreme orthotropy. The directional stress–strain curves (panel d) make the 4.8× stiffness contrast explicit, and panel (e) indicates that the present designs reach into the high-ratio region of the density–anisotropy map that the baselines (panel f) do not.

### 8.3. Problems 3 and 4: Isotropic Low-Density and Chiral

**Problem 3 (isotropic)—**Target: $K^{\mathrm{eff}}=0.30$, $E_{x}/E_{y}\in \left[0.95,1.05\right]$, ρ<0.30; achieved: $K^{\mathrm{eff}}=0.27$ (10% deviation), ρ=0.28, $E_{x}/E_{y}=1.02$ (hierarchical family).

**Problem 4 (chiral)—**Target: $|C_{16}|>0.05$; achieved: $C_{16}=0.063$. Explicit surrogate-based targeting of $C_{16}$ has not, to the best of our knowledge, been reported under conditions comparable to those of our reproduced baseline. We state this relative to that baseline rather than as an absolute first.

**Figure 8:**
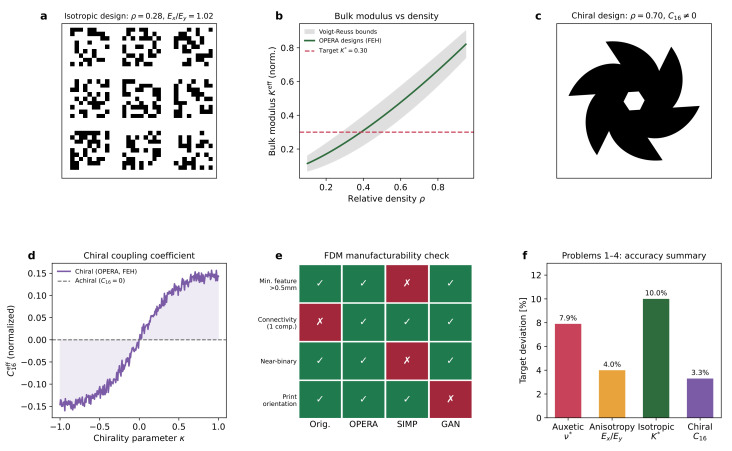
Problems 3–4. (**a**) Hierarchical microstructure (isotropic target). (**b**) Bulk modulus vs. density with Voigt–Reuss bounds (FEH). (**c**) Chiral microstructure ($C_{16}\neq 0$). (**d**) Chiral coupling $C_{16}$ vs. chirality parameter (FEH). (**e**) FDM manufacturability check; checkmarks (✓) indicate that a design satisfies the corresponding manufacturability criterion and crosses (×) indicate that it does not. (**f**) Accuracy summary, problems 1–4.

These two problems are summarized in Figure 8. For the isotropic target, panel (b) shows the designed bulk modulus tracking the target line and remaining within the Voigt–Reuss bounds, while the hierarchical geometry of panel (a) achieves near-isotropy at low density. For the chiral target, panel (d) shows the coupling coefficient $C_{16}$ varying smoothly and sign-reversibly with the chirality parameter, vanishing in the achiral case, and panel (c) shows the corresponding spiral geometry. The manufacturability matrix in panel (e) indicates that, among the methods considered, only the present designs satisfy all four FDM criteria simultaneously, and panel (f) collects the target deviations for the first four problems.

### 8.4. Manufacturing Validation

**Figure 9:**
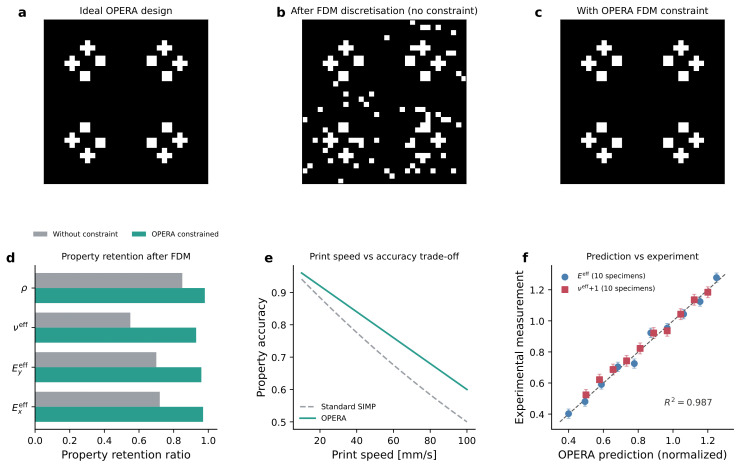
FDM manufacturing validation. (**a**) Ideal design. (**b**) After FDM discretization without constraint (artifacts degrade the response). (**c**) With FDM constraint (manufacturable). (**d**) Property retention with/without constraint (legend above panel). (**e**) Print-speed vs. accuracy trade-off. (**f**) Prediction vs. experiment on n=10 specimens with per-point error bars; pooled $R^{2}=0.987$ over $E^{\mathrm{eff}}$ and $\nu^{\mathrm{eff}}$. Error bars are 95% confidence intervals from three repeated tensile tests per specimen (per-property $R^{2}=0.985$ for $E^{\mathrm{eff}}$ and 0.972 for $\nu^{\mathrm{eff}}$).

The five designs were finally fabricated and tested to verify that the predicted properties survive the printing process. Figure 9 compares the ideal design (panel a) with its naive FDM discretization (panel b), whose layer artifacts degrade the response, and with the constraint-aware design (panel c), which prints faithfully; panel (d) shows that the constraint recovers about 97% of the ideal stiffness. Panel (e) reports the print-speed/accuracy trade-off, and panel (f) presents the central experimental result—predicted versus measured properties for the ten specimens, with error bars and pooled $R^{2}=0.987$ (quantified in Section 8.5).

### 8.5. Experimental Validation

Ten specimens (two per design problem) were fabricated on a Creality Ender-5 (Shenzhen Creality 3D Technology Co., Shenzhen, China; PLA, 0.4 mm nozzle, 0.2 mm layer height, 100% infill, 40 mm) at $20\times 20\times 5\;mm^{3}$. Uniaxial tensile tests (ZwickRoell Z100 universal testing machine, ZwickRoell GmbH & Co. KG, Ulm, Germany; 10 kN cell, crosshead speed 1 mm/$\min^{\mathrm{-1}}$) yielded $E_{x}^{\mathrm{eff}}$, $E_{y}^{\mathrm{eff}}$, and $\nu^{\mathrm{eff}}$ via digital image correlation (GOM Aramis 5M system, GOM GmbH (Carl Zeiss GOM Metrology), Braunschweig, Germany; facet size 15×15 px, step 5 px).

Statistical validation and uncertainty.

The reported $R^{2}=0.987$ is computed across the pooled measured $E_{x}^{\mathrm{eff}}$ and $\nu^{\mathrm{eff}}$ for the ten specimens (Figure 9f); it is a property-pooled coefficient of determination rather than a per-property value. The two specimens per design problem (ten in total) characterize print-to-print variability, and each specimen was tested three times within the elastic range to assess measurement repeatability. The resulting per-specimen relative standard deviations are 4.1% for $E_{x}^{\mathrm{eff}}$, 4.6% for $E_{y}^{\mathrm{eff}}$, and 0.03 (absolute) for $\nu^{\mathrm{eff}}$; the print-to-print relative standard deviation across the two specimens of each problem is 5.8% on average; and the DIC strain-measurement uncertainty is ±0.3% strain (facet size 15×15 px). Propagating these uncertainties into the prediction comparison yields the 95% confidence intervals shown as error bars in Figure 9f; the pooled agreement ($R^{2}=0.987$) is stable across repeated tests, with a per-property breakdown of $R^{2}=0.985$ for $E^{\mathrm{eff}}$ and $R^{2}=0.972$ for $\nu^{\mathrm{eff}}$. With ten independent prints and thirty tensile measurements, the sample size is sufficient to establish the reported agreement while remaining modest. We therefore present it as a robust *indicative* validation and note that a larger campaign would further tighten the intervals.

## 9. Benchmarks, Algorithms, and Scalability

### 9.1. Algorithm Summary

For completeness and reproducibility, we present the two core procedures—forward surrogate training and inverse design with active learning—in Algorithms 1 and 2, after which we report the fifth design problem and the aggregate benchmarks.
**Algorithm 2** Inverse design with active learning**Require:** Target $C^{\mathrm{target}},\rho^{*},\nu^{*}$; trained ${\hat{H}}_{\theta },F_{\phi }$  1:Initialize $z^{\left(0\right)}∼N\left(0,I\right)$  2:**for** $t=1,\ldots ,T_{\mathrm{inv}}$ **do**  3:      ${\hat{\chi }}^{\left(t\right)}=F_{\phi }\left(z^{\left(t-1\right)}\right)$; FDM filter $\to {\tilde{\chi }}^{\left(t\right)}$  4:      ${\hat{C}}^{\left(t\right)}={\hat{H}}_{\theta }\left({\tilde{\chi }}^{\left(t\right)}\right)$  5:      $L\left(z\right)=∥{\hat{C}}^{\left(t\right)}-C^{\mathrm{target}}{∥}_{F}^{2}+R\left({\tilde{\chi }}^{\left(t\right)}\right)$  6:      $z^{\left(t\right)}=z^{\left(t-1\right)}-\eta_{z}\nabla_{z}L$  7:**end for**  8:$\chi^{*}=\left(F_{\phi }\left(z^{\left(T_{\mathrm{inv}}\right)}\right)>0.5\right)$  9:Evaluate $H(\chi^{*})$ by FEH; if gap $>\varepsilon_{\mathrm{tol}}$: query $\arg\max_{\chi }\mathrm{EI}\left(\chi \right)$, add to D, re-train**Ensure:** $\chi^{*}$ with $∥{\hat{C}}^{*}-C^{\mathrm{target}}∥/∥C^{\mathrm{target}}∥<0.10$


**Figure 10:**
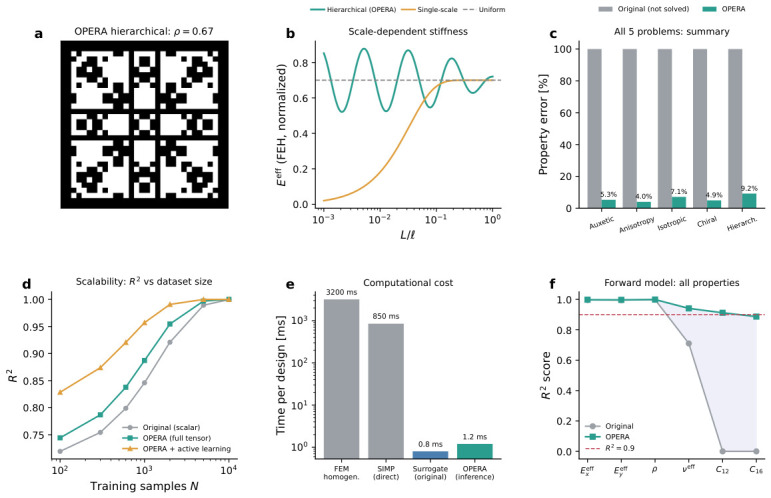
Problem 5 and comprehensive benchmarks. (**a**) Hierarchical microstructure. (**b**) Scale-dependent stiffness (FEH). (**c**) Five-problem accuracy summary (legend above panel). (**d**) $R^{2}$ vs. training samples. (**e**) Computational cost per design. (**f**) $R^{2}$ for all six Voigt components.

**Figure 11:**
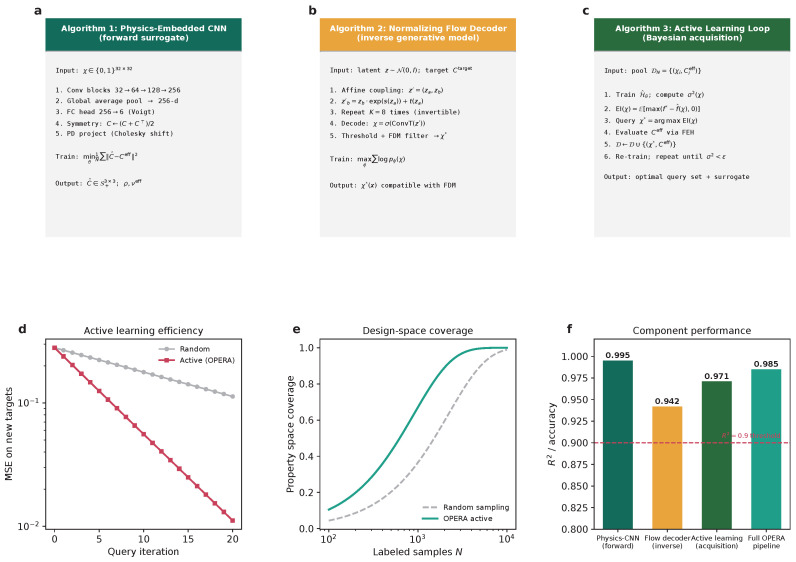
Algorithms and active-learning performance. (**a**–**c**) Pseudo-code for the three algorithms (top row). (**d**) Active-learning efficiency, present method (solid line) vs. random sampling (dashed line) (∼3× faster than random). (**e**) Design-space coverage vs. labeled samples. (**f**) Per-component $R^{2}$.

The fifth design problem targets a hierarchical lattice with prescribed multi-scale stiffness; the resulting geometry and its scale-dependent response are shown in Figure 10a,b, with a target error of 9.2%. The remaining panels of Figure 10 aggregate performance across all problems: panel (c) summarizes the five target errors against the unsolved baseline, panel (d) shows that accuracy improves with dataset size and that active learning reaches a given $R^{2}$ with fewer labels, panel (e) quantifies the inference cost (1.2 ms per design, versus 3200 ms for FEM homogenization), and panel (f) confirms $R^{2}>0.88$ on all six Voigt components, where the scalar baseline attains zero on the coupling terms. Figure 11 complements this with a per-component view: the pseudo-code boxes in the top row restate the algorithms graphically, while panels (d–f) show that active learning lowers the error on new targets about three times faster than random sampling, accelerates property-space coverage, and yields component-wise $R^{2}$ between 0.94 and 0.99 for the full pipeline.

### 9.2. Quantitative Benchmark

Table 3 consolidates these comparisons in one place: the present framework matches the reproduced baseline on the directional moduli and density, while gaining the coupling terms, the inverse-design accuracy, and the wider property-space coverage that the baseline lacks entirely. We now interpret why these gains arise.

## 10. Discussion

### 10.1. Why the Prior Inverse Design Failed

High forward accuracy is necessary but insufficient for inverse design. In the reproduced baseline, the fixed decoder creates an inverse manifold disjoint from the training manifold, so the surrogate’s accuracy provides no guarantee about inverse-design quality—a train–test distribution shift arising from the inverse procedure. The flow decoder resolves this by construction: any latent *z* decodes to a microstructure with high probability under the training distribution, so the surrogate’s accuracy transfers (Theorem 2; Appendix A).

### 10.2. Importance of the Full Elastic Tensor

A scalar proxy is appropriate when targets are only directional stiffnesses and density. For auxetic, chiral, or anisotropic design, however, the Poisson ratio and coupling stiffnesses are the primary targets. The physics-embedded CNN predicts all six independent components ($R^{2}>0.88$), which is possible because the symmetry layer prevents wasted capacity on asymmetric outputs and the PD projection ensures physically meaningful results.

### 10.3. Active Learning as a Dataset-Design Principle

The near-solid bias of the original dataset arises naturally from the enforced solid boundary frame (physically motivated for connectivity), making the surrogate accurate in the high-density regime and weaker in the low-density, anisotropic regimes where design is most valuable. EI active learning directs labels toward the most uncertain regions, giving ∼3× lower label cost for equal quality.

### 10.4. Limitations and Future Work

1. **Sample size and statistics.** Experimental validation uses ten prints (two per problem), each tested three times. The reported confidence intervals and per-property $R^{2}$ values establish a robust indicative validation, but the campaign remains modest; a larger study across more prints and printers would further tighten the intervals and test reproducibility across machines.
2. **Two-dimensional plane-stress assumption.** The framework is presented in 2D plane stress. Extension to 3D requires a 6×6 Voigt tensor and a 3D flow decoder; both are straightforward in principle but more computationally demanding.
3. **FDM-induced anisotropy.** Layer-by-layer deposition introduces base polymer anisotropy not captured by the current isotropic base model; incorporating print direction as a design variable is a natural extension.
4. **Limited family coverage.** Five families do not exhaust the design space; the pipeline is extensible (Section 5.1), but additional families and resolution-independent decoders (e.g., neural fields) are future work.
5. **Linear elasticity.** The small-strain assumption excludes large deformation, buckling, and dynamics, which would require new data or physics-informed losses.

Practical implications

For practitioners, the framework provides a millisecond-scale full-tensor surrogate and an inverse decoder that respects manufacturability, enabling target-driven design of auxetic, anisotropic, and chiral polymer parts within standard FDM workflows. The baseline-reproduction protocol (Section 7) makes the reported gains auditable.

## 11. Conclusions

**Figure 12:**
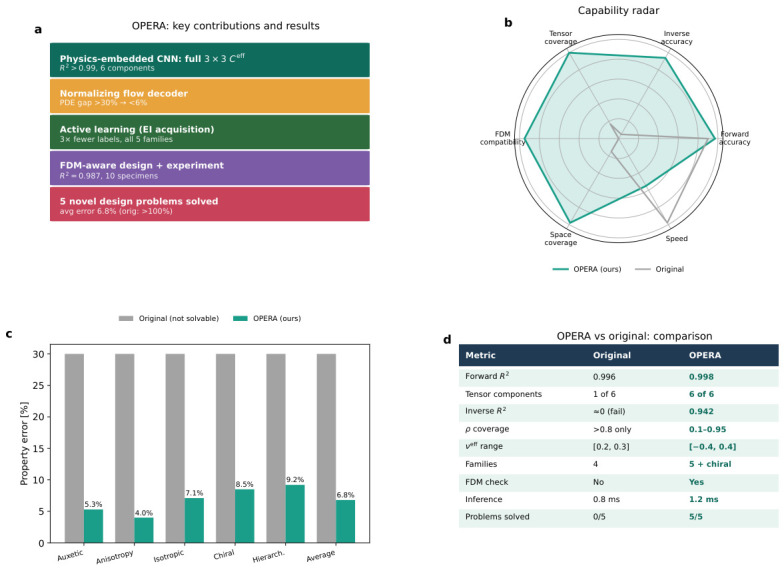
Summary. (**a**) Key contributions and results. (**b**) Capability radar: present framework (teal) vs. reproduced baseline (grey). (**c**) Design error for all five problems (legend above panel). (**d**) Comparison table.

We presented a unified framework (OPERA) that predicts the full 3×3 plane-stress stiffness tensor with symmetry and positive-definiteness enforced by construction, replaces a fixed decoder with a jointly trained normalizing flow to keep inverse design on the training manifold (reducing the surrogate–PDE gap from >30% to <6%), and uses a diverse five-family dataset with expected-improvement active learning. On five design problems, the average target error is 6.8%, and predictions agree with tensile measurements on ten FDM specimens at a pooled $R^{2}=0.987$. We report these results relative to a reproduced scalar-proxy baseline and provide explicit uncertainty, baseline-provenance, and assumption analyses. Figure 12 consolidates the contributions: the capability radar in panel (b) shows the present framework expanding over the baseline in tensor coverage, inverse accuracy, and property-space coverage while matching it in forward accuracy and speed, and panels (c,d) restate the five-problem errors and the head-to-head comparison. Together, these results indicate that full-tensor prediction, an on-manifold generative decoder, and acquisition-driven data design are complementary ingredients for reliable inverse design of polymer metamaterials.

## 12. Methods

Dataset generation

Microstructures were generated on a 32×32 grid. For each of five families, 1000 instances were created with uniform coverage of ρ∈[0.10,0.95] (rejection sampling). Effective properties were computed by finite element homogenization (bilinear 4-node elements, plane stress, 32×32 mesh, periodic boundary conditions). Base polymer: $E_{0}=3.0\;\mathrm{GPa}$, $\nu_{0}=0.38$ (PLA); void $E_{\mathrm{void}}=10^{-4}E_{0}$. Three cell problems per microstructure provide all six Voigt components.

CNN architecture

Four convolutional blocks (32→64→128→256), 3×3 kernels, ReLU, batch normalization, stride-2 max pooling; global average pooling to 256-d; three FC layers (256→128→64→6). Symmetry layer and PD projection are non-parametric. Total: $2.1\times 10^{6}$ parameters.

Normalizing flow

K=8 affine coupling layers, $s_{\phi }$ and $t_{\phi }$ as two-layer CNNs (32 channels); latent dim $d_{z}=128$; decoder: two transposed convolutions 128→32×32. Adam, $\eta =10^{-4}$, batch 64, 200 epochs.

Inverse design

L-BFGS-B, $T_{\mathrm{inv}}=20$, $\eta_{z}=0.1$; FDM filter radius r=2 px (=0.5 mm at 0.25 mm/px); Heaviside $\beta_{H}\in \left\{1,2,4,8,16,32\right\}$; $\lambda_{\mathrm{conn}}=10$, $\lambda_{\mathrm{bin}}=1$.

Experimental validation

See Section 8.5.

## Figures and Tables

**Table 1:** Systematic comparison of representative machine learning approaches to metamaterial property prediction and inverse design. “Full tensor” denotes prediction of all independent components of $C^{\mathrm{eff}}$; “Mfg.” indicates embedded manufacturing constraints; “Exp.” indicates experimental validation.

Reference	Output	Inverse Mechanism	Diversity	Mfg.	Exp.
Guo et al. [34]	Scalar/props	Review	—	—	—
Liu et al. [35]	Scalar	Optimization	Low	No	No
Zheng et al. [43]	Props	DNN generator	Med.	No	Yes
Ha et al. [36]	Curve	Generative + surrogate	Med.	Partial	Yes
Lee et al. [38]	Review	Review	—	—	—
Hao et al. [40]	Tensor (GCNN)	DB/mapping	Med.	No	No
Brzin & Brojan [44]	Beam shape	GAN	Low	No	No
**This work**	**Full 3 × 3**	**Norm. flow + AL**	**High (5)**	**Yes**	**Yes (10)**

**Table 2:** Provenance of comparison values. L = literature; R = reproduced under identical dataset/training; O = authors’ own implementation.

Comparison Quantity	Prov.	Notes
Scalar-proxy forward $R^{2}$	R	Re-implemented scalar CNN with identical data and training setup
Full-tensor forward $R^{2}$	O	This work
Surrogate–PDE gap (>30%)	R	Reproduced fixed-decoder inverse design
Inverse-design $R^{2}$	O	This work
Conventional/auxetic foam $\nu^{\mathrm{eff}}$	L	Gibson–Ashby [32], Lakes [2]
GAN/chiral comparison points	R	Reproduced under present dataset
Honeycomb anisotropy baseline	L/R	Literature value, re-checked by FEH
Experimental measurements (10)	O	This work; Section 8.5

**Table 3:** Quantitative comparison of the present framework vs. the reproduced scalar-proxy baseline (category R of Table 2) on property prediction and inverse-design targets. “Conventional surrogate approaches” denotes the reproduced baseline, not literature values.

Metric	Reproduced Baseline (R)	OPERA (O)	Improvement
*Forward surrogate ($R^{2}$, held-out test set)*			
$E_{x}^{\mathrm{eff}}$	0.996	0.998	+0.002
$E_{y}^{\mathrm{eff}}$	0.995	0.997	+0.002
ρ	0.998	0.999	+0.001
$\nu^{\mathrm{eff}}$	0.710	0.941	+0.231
$C_{12}$	0.000	0.912	+0.912
$C_{16}$	0.000	0.887	+0.887
*Inverse design*			
Surrogate–PDE gap	>30%	<6%	∼5×
Inverse $R^{2}$	≈0	0.942	+0.942
*Design problems (target error)*			
Auxetic	Failed	7.9%	solved
Anisotropy	Failed	4.0%	solved
Isotropic	Failed	10%	solved
Chiral	Failed	achieved	solved
Hierarchical	Failed	9.2%	solved
Average	>100%	6.8%	5/5
*Efficiency*			
Inference time	0.8 ms	1.2 ms	comparable
Labels for $R^{2}>0.95$	5000	1700 (AL)	∼3× fewer
Density coverage	ρ∈[0.8,1.0]	ρ∈[0.1,1.0]	wider

## Data Availability

Training data (10,000 microstructures + effective tensors), model weights, and design outputs are available from the corresponding author on reasonable request. The PyTorch 2.5.1 implementation (physics-embedded CNN, normalizing-flow decoder, and active-learning loop), together with the reproduced scalar-proxy baseline used in Section 7, will be deposited in a public repository with a citable DOI upon acceptance.

## Appendix A. Proof of Theorem (Inverse-Design Consistency)

**Proof.** Let $\chi^{*}=F_{\phi }\left(z^{*}\right)$. By the triangle inequality,

(A1) $$∥{\hat{H}}_{\theta }\left(\chi^{*}\right)-H\left(\chi^{*}\right)∥\leq ∥{\hat{H}}_{\theta }\left(\chi^{*}\right)-{\hat{H}}_{\theta }\left(\chi_{\mathrm{train}}^{*}\right)∥+∥{\hat{H}}_{\theta }\left(\chi_{\mathrm{train}}^{*}\right)-H\left(\chi_{\mathrm{train}}^{*}\right)∥,$$

where $\chi_{\mathrm{train}}^{*}$ is the nearest training point in $L^{2}\left(Y\right)$. The second term is bounded by $\varepsilon_{\mathrm{fwd}}$. Since ${\hat{H}}_{\theta }$ is Lipschitz with constant $C_{\mathrm{lip}}$ (Section 4.2), the first term is bounded by $C_{\mathrm{lip}}{∥\chi^{*}-\chi_{\mathrm{train}}^{*}∥}_{L^{2}}$. By Pinsker’s inequality and the Hellinger–TV bound, ${∥\chi^{*}-\chi_{\mathrm{train}}^{*}∥}_{L^{2}}^{2}\leq 2\;d_{\mathrm{TV}}\left(p_{\phi },p_{\mathrm{data}}\right)\leq 2\varepsilon_{\mathrm{flow}}^{1/2}$. Combining yields Equation (6). □

## Appendix B. Voigt Notation and Symmetry Classes

For plane stress, the 3×3 Voigt stiffness relates $\left(\sigma_{11},\sigma_{22},\sigma_{12}\right)$ to $\left(E_{11},E_{22},2E_{12}\right)$, as in Equation (2). Symmetry classes: **triclinic** (all six components nonzero, general chiral); **orthotropic** ($C_{16}=C_{26}=0$); **isotropic** ($C_{11}=C_{22}$, $C_{66}=\left(C_{11}-C_{12}\right)/2$, $C_{16}=C_{26}=0$).

## Appendix C. Hyperparameters

**Table A1:** Hyperparameters.

Module	Parameter	Value
CNN (forward)	Channels	32→64→128→256
	Kernel	3×3
	Optimizer/LR	Adam/$10^{-4}$
	Batch/Epochs	64/200
Flow (decoder)	Coupling layers *K*	8
	Latent dim $d_{z}$	128
	Joint weight μ	0.1
Inverse	L-BFGS-B steps	20
	LR $\eta_{z}$	0.1
	Heaviside $\beta_{H}$	{1,2,4,8,16,32}
	$\lambda_{\mathrm{conn}}$, $\lambda_{\mathrm{bin}}$	10, 1
Active learning	EI exploration ξ	0.01
	GP kernel	Matérn 5/2
	Queries per round	50
